# Insight into transcription factor gene duplication from *Caenorhabditis elegans *Promoterome-driven expression patterns

**DOI:** 10.1186/1471-2164-8-27

**Published:** 2007-01-23

**Authors:** John S Reece-Hoyes, Jane Shingles, Denis Dupuy, Christian A Grove, Albertha JM Walhout, Marc Vidal, Ian A Hope

**Affiliations:** 1 Institute of Integrative and Comparative Biology, Faculty of Biological Sciences, University of Leeds, Clarendon Way, Leeds, LS2 9JT, West Yorkshire, UK; 2 Center for Cancer Systems Biology (CCSB), and Department of Cancer Biology, Dana-Farber Cancer Institute and Department of Genetics, Harvard Medical School, 44 Binney Street, Boston, Massachusetts 02115, USA; 3 Program in Gene Function and Expression and Program in Molecular Medicine, University of Massachusetts Medical School, Worcester, 364 Plantation Street, Lazare Research Building, Room 605, MA 01605, USA

## Abstract

**Background:**

The *C. elegans *Promoterome is a powerful resource for revealing the regulatory mechanisms by which transcription is controlled pan-genomically. Transcription factors will form the core of any systems biology model of genome control and therefore the promoter activity of Promoterome inserts for *C. elegans *transcription factor genes was examined, *in vivo*, with a reporter gene approach.

**Results:**

Transgenic *C. elegans *strains were generated for 366 transcription factor promoter/*gfp *reporter gene fusions. GFP distributions were determined, and then summarized with reference to developmental stage and cell type. Reliability of these data was demonstrated by comparison to previously described gene product distributions. A detailed consideration of the results for one *C. elegans *transcription factor gene family, the Six family, comprising *ceh-32*, *ceh-33*, *ceh-34 *and *unc-39 *illustrates the value of these analyses. The high proportion of Promoterome reporter fusions that drove GFP expression, compared to previous studies, led to the hypothesis that transcription factor genes might be involved in local gene duplication events less frequently than other genes. Comparison of transcription factor genes of *C. elegans *and *Caenorhabditis briggsae *was therefore carried out and revealed very few examples of functional gene duplication since the divergence of these species for most, but not all, transcription factor gene families.

**Conclusion:**

Examining reporter expression patterns for hundreds of promoters informs, and thereby improves, interpretation of this data type. Genes encoding transcription factors involved in intrinsic developmental control processes appear acutely sensitive to changes in gene dosage through local gene duplication, on an evolutionary time scale.

## Background

A proper understanding of complex biological processes may well require a complete knowledge of all the contributing elements, an appreciation of the operation of the entire system. However, the precise quantities of and the range of gene products constituting an individual cell may well be unique to each cell generated during each animal's development. Each cell will occupy at least a subtly unique environment within the body and will have followed at least a subtly unique developmental history as a consequence of the stochastic nature of molecular level events. A primary determinant of the spectrum of gene products in each cell will be the spectrum of sequence specific transcription factors that have driven expression of the genome during that cell's developmental history. The scale of this problem, which needs to be addressed to fully understand an animal's development, would be dramatically reduced by using a species in which development, at least down to the level of individual cells, is invariant. These considerations make the free-living soil nematode *Caenorhabditis elegans *an excellent choice for systems biology approaches [1]. Furthermore, the remarkable conservation of biological processes, at the molecular genetic level, means that findings with this animal are likely to be widely relevant.

The essentially invariant and fully described developmental cell lineage of *C. elegans *[2] provides a framework within which the systems biology of this animal can be understood and could be depicted. *C. elegans *was also the first animal to have its genome sequenced [3] and this has lead to a plethora of genome-wide analyses using various technologies to study gene function [4,5]. Maps of gene expression, in terms of the cell lineage, will be essential to integration of these datasets.

Typically, *C. elegans *gene expression pattern data are gathered, in terms of the cell lineage, using strains transformed with reporter gene fusions [6,7]. This approach combines the highest resolution with the efficiency necessary for high throughput studies. A genomic DNA segment containing a *C. elegans *gene's regulatory regions is joined to a reporter gene and, after the fusion gene is introduced into the genome by transformation, the distribution of the reporter gene product can be readily determined, *in situ*, in large numbers of individuals of the transgenic strains. Whether the fusion gene is intended to report on just a gene's promoter activity, by driving reporter expression with a DNA fragment from immediately upstream of the gene start [8], or to report on the distribution of the gene product, by seamless insertion of the reporter into the protein coding region within a large multi-gene DNA fragment [9], there can be questions about how accurately the observations reflect expression of the endogenous gene. Have all the relevant regulatory elements really been included in the fusion? Furthermore, for the majority of transgenic *C. elegans *strains the introduced DNA is maintained as an extrachromosomal array, with hundreds of copies of the fusion gene all joined together in each cell, not even integrated into a proper *C. elegans *chromosome [10]. Does this unnatural environment also modify the expression pattern of the fusion gene?

Our attention has been directed at the promoter regions of *C. elegans *transcription factor genes. The *C. elegans *genome is more densely packed than most other animal genomes with over half the genome found in transcribed regions [3]. Expression is initiated by transcription driven from the promoter located immediately upstream of the transcription unit. Regulatory elements through which the activity of a promoter is controlled are generally found to be concentrated in the immediately upstream intergenic region (e.g. [11,12]), perhaps a reflection of the high gene density in this species. Therefore these regions have been the specific subject of a large-scale cloning project, generating the Promoterome [8]. *C. elegans *promoters were cloned using the MultiSite Gateway system [13] so that exactly the same DNA fragments could be subject to multiple large scale analyses, including reporter fusion [8] and protein::DNA interaction [14] assays. Transcriptional regulatory elements and the transcription factors that bind to them will lie at the core of any model of the control of expression of the genome and so we chose to start our analysis with Promoterome clones for transcription factor genes [15].

Promoterome inserts for *C. elegans *transcription factor genes were transferred by Gateway recombination into a reporter vector for fusion to *gfp *[8]. The *C. elegans unc-119 *gene was included in the vector backbone to allow selection following transformation by microprojectile bombardment [16]. This procedure yields transgenic lines with the transforming DNA inserted at low copy number into *C. elegans *chromosomes, as well as transgenic lines with the transforming DNA present in extrachromosomal arrays, the typical product of transformation by the more traditional microinjection method [10]. The GFP expression, in all transgenic lines established, was examined through all stages of development by fluorescence microscopy. The reliability of the reporter expression pattern data generated was evaluated and conclusions were drawn concerning transcription factor evolution.

## Results and discussion

### *C. elegans *transcription factor gene promoter/reporter gene fusions

A recent comprehensive analysis of the *C. elegans *genome using a combination of computational and manual interrogation methods compiled a list of 934 putative transcription factor genes, wTF2.0 [15] and this formed the starting point of our investigation. Expression patterns were determined for 366 of the 640 Promoterome clones so far generated for these genes.

The Promoterome clones contain DNA fragments of varying length but all ending at the start of the protein coding region for each gene. The translational initiation codon was selected as the downstream end point in the cloned DNA fragment of each promoterome clone, as trans-splicing means the transcriptional start is known for few *C. elegans *genes. However, 5' UTRs of *C. elegans *transcripts tend to be relatively short [17] and typically the entire intergenic region was targeted. Where the intergenic region is less than 300 bp, a 300 bp fragment extending into the coding region of the upstream gene was cloned. Where the intergenic region is more than 2000 bp, the 2000 bp immediately upstream of the translational initiation codon was targeted. Where initial cloning was unsuccessful, 1500 bp fragments were cloned instead.

Many (258) of the cloned promoters assayed here were present in the original Promoterome library, Version 1.0 [8], which had not been specifically targeted at transcription factor genes. A subsequent large scale cloning exercise, directed at wTF2.0, yielded another 351 promoter clones. In addition, a further 31 promoters were cloned specifically to identify overlaps in expression domains that could reveal potential functional overlap among transcription factor family members: both synergistic functional overlap, e.g. among families of transcription factors known to heterodimerise (bHLH, homeodomain, paired domain) and redundant functional overlap.

Promoterome clones were used to generate promoter::*gfp *fusions by Gateway recombination. Although some transformation by microinjection was performed (20 genes), plasmids bearing the fusions were mostly used in transformation of *C. elegans *by microprojectile bombardment (350 genes, 4 by both techniques) [see additional file 1]. High-throughput microprojectile bombardment requires a continuous supply of *unc-119 *mutant *C. elegans*, on a large scale, and for this we devised a cyclical liquid media protocol. For each reporter fusion, up to eight independent transgenic lines were created. For each line, the rate of transmission of the transgene, both meiotically, between generations, and mitotically, during development of individual animals, and the level and pattern of GFP expression were assessed. Unlike microinjection, bombardment frequently results in transformed lines that have the transgene stably integrated into the genome. Approximately one in every eight lines generated in this study appeared to have the introduced DNA chromosomally integrated, less than the 25% rate reported by others [16]. Typically, after the GFP expression pattern was documented, three transgenic strains were retained for each promoter-reporter fusion and are available for further studies.

The GFP expression patterns driven by Promoterome inserts for 366 *C. elegans *transcription factor genes have been determined. Attention was concentrated on the smaller transcription factor families, such as homeodomain and bHLH proteins. More than 2500 independent transgenic lines were generated and examined, and over 1000 of these have been retained for future use.

### Expression pattern characterization

In each transgenic line established, the GFP expression pattern was observed in intact hermaphrodites from a mixed age population with worms of all developmental stages. Complete transparency throughout this animal's development means that GFP can be readily observed in any cell in whole mounts. Thousands of individuals were given a cursory examination with a few individuals studied more carefully, and at higher magnification. Each expression pattern was determined with respect to early, mid and late embryogenesis, through early, mid and late larval stages to fully developed adult. Depending on the nature of the pattern, expression was determined to tissue type or down to the specific cells [See additional file 1]. Illustrative micrographs of each expression pattern, along with text descriptions, were entered into a web-accessible database [18].

For any given reporter gene fusion, the same expression pattern was observed consistently in all independent transgenic lines that showed GFP expression, whether generated by microinjection or bombardment. Expression intensity did vary considerably between lines generated by bombardment, more than seen with transformation by microinjection, and this is presumably the reason why expression was not detected in all lines for functional fusions, particularly for those with weak promoters. Nevertheless, for most reporter gene fusions assayed that drove GFP expression, almost all or all lines showed expression. The expression pattern was not accepted as genuine unless that expression pattern was seen in at least two independent lines. Lines with the transforming DNA integrated into a chromosome (by non-homologous insertion) typically had lower expression levels, presumably due to the lower copy-number of the reporter fusion [16], but complete expression patterns were then seen in all individuals at the appropriate stage of development. In lines with the transforming DNA present in extrachromosomal arrays, and not integrated into a chromosome, expression could be very strong. Extrachromosomal arrays generated by microinjection contain hundreds of copies of the reporter fusion gene [10] but copy number was not determined for any of the lines generated here. Extrachromosomal arrays are not reliably transmitted to all cells during an individual's development or to all an individual's progeny, so not all individuals in populations of such lines contain the transgene and each individual that does is a mosaic of cells with and without the transgene. For these mosaic lines, therefore, a larger number of animals were examined closely so that each component of the expression pattern was observed and the complete expression pattern could be appreciated. Interestingly, in comparison to the corresponding extrachromosomal array lines, no extraneous expression was observed in any of the 135 chromosomally integrated lines suggesting that the position of insertion into the genome had no effect on reporter expression. This would be consistent with the apparently tight organization of genetic units in *C. elegans*, but it is still remarkable that no examples of such influence were observed in such a large number of integration events. Within any given expression pattern, expression intensity varies among tissues. While this observation may be due to some cell types being larger or more obvious than others (e.g. excretory cell versus a single neuron), this will also reflect differences in the endogenous cell-specific activity of the promoter between cells.

A summary of the expression pattern data gathered in this study is provided in Table 1. GFP expression was observed, in hermaphrodites under standard laboratory conditions, for 92% (335 of 366) of the promoter reporter gene fusions assayed. The 31 Promoterome inserts that failed to drive GFP expression may be from inactive genes, may contain promoters with activity that is male-specific, only induced under specific environmental conditions, or too weak to be observed by the method followed, or the gene model used in selecting the promoter region may be wrong. Three of these Promoterome inserts are from downstream genes in operons, so the expression of these genes appears totally dependent upon promoters of upstream genes in the operon, that were not within the DNA fragment assayed.

Most of the expression patterns driven by the *C. elegans *transcription factor gene promoters assayed showed a restricted distribution with only 31 (8%) of the reporter gene fusions driving GFP expression constitutively. Every *C. elegans *tissue-type was represented amongst the restricted GFP expression patterns with the exception of the germ line. Germ line expression is generally only seen in transgenic lines of *C. elegans *in which the transforming DNA is chromosomally integrated [19] and such a line has not been generated for all the promoter reporter fusion genes assayed. However, few transcription factors, sequence specific DNA binding proteins that typically regulate transcription of target genes to control development or tissue specific responses to environmental conditions, would be expected to be expressed either in the germ line or constitutively.

The majority of *C. elegans *transcription factor promoter fragments assayed in this study drive reporter expression in the nervous system (246 promoters, 67%). The nervous system constitutes 302 of the 959 somatic cells in the adult hermaphrodite and 118 nerve cell classes can be distinguished anatomically [20]. Transcription factors are expected to have a major role in determining cell identities within this complex tissue and, through evolution, many transcription factors involved in other aspects of development would have been recruited to distinguish or modify nerve cell fates. Over half of the somatic cells and almost all the tissue types in *C. elegans *are generated during embryogenesis and so it is not surprising that 82% (299) of the promoter reporter fusions are active during embryonic stages of development. These general statistics are entirely consistent with expectations, if the Promoterome inserts are indeed providing an accurate reflection of the expression of the endogenous genes.

### Reliability of reporter expression pattern data

Expression patterns based on typical *C. elegans *reporter fusions are subject to the caveat of whether DNA segments fused to the reporter contain all the elements required to fully replicate the distribution of the endogenous gene product. Even for complex reporter fusions, when the reporter gene is inserted into a cloned DNA fragment maintaining all in the vicinity of the target gene, a more distantly located element could always exist that is essential for the correct pattern of transcription [21]. Simple reporter fusions, made using only the upstream region, will, of course, lack all post-transcriptional aspects of regulation, such as that mediated by microRNAs, but may also lack transcriptional regulatory elements located in introns, in exons or downstream of the target gene. The compactness of the *C. elegans *genome [3], however, may make it more likely than for some other animals that a DNA fragment from immediately upstream of the protein-coding region will contain the promoter and most or even all of the required transcriptional control elements.

For some *C. elegans *transcription factor genes, intergenic DNA fragments of the size cloned in the Promoterome resource [8], 1.5 to 2.0 kb, are enough to drive the complete endogenous expression pattern. For example, deletion studies using the promoters of *ceh-16 *[22] and *ceh-22 *[23] revealed that only 1 kb and 1.9 kb of upstream region, respectively, is required for reporter gene expression to match the pattern revealed using a specific antibody to the gene product. However, for other genes such small upstream regions are not sufficient: *egl-5 *needs 8 kb of upstream region plus elements downstream from the start of the protein coding region [24] and *lin-39 *needs 10.5 kb of upstream region plus the first intron, although the region downstream of the protein coding region appears dispensable [25].

To assess the reliability of expression patterns driven by Promoterome inserts we compared our data to published expression patterns generated by either immunofluorescence microscopy or *in situ *hybridization [See additional file 2] which reveal gene product, protein or mRNA, distributions directly. Such data exist for 40 of the 366 *C. elegans *transcription factors we assayed. Comparing these datasets is not straightforward because the expression pattern descriptions can be taken to different resolutions by the different methods. Furthermore, the descriptions are referring to different aspects of the expression pattern: Promoterome reporter gene fusions display transcriptional activity while transcript or protein distributions are subject to post-transcriptional events such as stability differences and protein trafficking. Nevertheless, nearly half of the Promoterome-driven expression patterns (17 of 40) appeared to recapitulate the published expression descriptions indicating that these fragments contain all the regulatory elements required to direct the endogenous transcription pattern. Almost all the remainder (19 of 23) appeared to include expression domains contained within the published descriptions, suggesting that the reporter pattern was incomplete but that the Promoterome insert assayed was accurately driving at least one component of the endogenous gene's expression and contains all the regulatory elements essential for this component. Obviously, a missing component in the expression pattern could be because the DNA fragment assayed in the reporter fusion is missing a binding site for a transcriptional activator of relevance to the promoter's activity. However, a non-misleading reason for an incomplete pattern for the reporter fusion is that only one Promoterome insert was assayed for each gene and alternative promoters could drive expression of the missing components. Five of these Promoterome clones do indeed target genes already known to have alternate start codons.

Only four of the forty Promoterome driven expression patterns used in the comparison showed no overlap with the antibody or mRNA *in situ *data. For three of these the previous data concerned expression in the germ line (or the very early embryo, which is typically derived from transcripts present in the germ line) and the difficulty of driving reporter expression in the germ line is well known [19]. Germ line expression of reporters appears favoured by chromosomal integration of fusion genes and no chromosomally integrated lines were generated for Promoterome fusions for one of these genes, *mep-1*. The total lack of detectable reporter expression in the chromosomally-integrated lines that were generated for Promoterome fusions for the other two genes, *mex-5 *and *oma-2*, not even revealing the expression seen in the corresponding extrachromosomal array bearing lines, may mean that expression in these integrated lines was accurately reflecting the endogenous promoter activity but the low copy number and feeble promoters made this expression too weak to be observed. For *unc-86*, the fourth gene for which the Promoterome expression pattern showed no overlap with the immunomicroscopy or mRNA *in situ *data, there was simply no reporter expression observed. However, the predicted translation initiation codon, as presented in WormBase and used in design of the Promoterome insert, does not correspond with the start of the protein coding region implied by EST data or gene prediction programmes, and therefore the *unc-86 *promoter had probably not been fused properly to the reporter.

Most of the Promoterome driven expression patterns (33 of 40) did include additional components in comparison to the immunofluorescence microscopy or *in situ *hybridization data but this is not as misleading as the statistics might suggest. The most obvious reason explaining these extra domains would be that the Promoterome insert lacks regulatory elements that are required for binding of transcription factors needed to silence expression otherwise driven in these domains by transcriptional activators that bind to the promoter region. However, a complication with reporter-based assays in *C. elegans *is the anecdotal evidence that vectors used for reporter fusions contain cryptic enhancer elements that can drive spurious expression, for example in the pharynx [26] or posterior intestine [27]. Artifactual pharyngeal expression may be revealed when *C. elegans *promoters are subject to deletion analysis, as for example with the *ges-1 *promoter [28]. Posterior intestine expression has been linked to the *unc-54 *3'UTR commonly used for transcript stability in *C. elegans *reporter constructs [27]. All the promoters assayed in this study were cloned into the same Gateway GFP-reporter destination vector, which does include the *unc-54 *3'UTR. *C. elegans *transformed with an empty Gateway GFP-reporter vector, lacking an insert, display no GFP expression, despite there still being DNA, vector DNA, upstream from the reporter. This reveals the need for a DNA fragment with at least basal promoter activity even for spurious expression of the reporter. However, many of the promoter::GFP fusions we assayed drove expression in the posterior intestine, pharynx and anterior head muscles (and less-frequently other body wall muscles) that we speculate may be artifactual. While this expression cannot be simply dismissed, as some genes will be expressed in these tissues, these components in the descriptions must be considered as potentially irrelevant to the endogenous gene's promoter activity. Such artifacts are more obvious when assaying very large numbers of promoters in the same vector, but mean caution must be exercised when *C. elegans *genes are studied individually with reporter technology.

If the trends observed in this comparison with prior data were applicable to *C. elegans *genes more generally, beyond our selected set of transcription factor genes, then 90% of Promoterome inserts will drive expression in endogenously significant domains, with almost half fully replicating the complete expression pattern. The Promoterome would then be a particularly valuable resource for investigating *C. elegans *promoter activity, especially for identifying DNA elements required for specific expression pattern components and the transcription factors that bind them [8]. Of course, other techniques for examining gene expression patterns may not report accurately on a promoter's transcriptional activity because of the influence of posttranscriptional events.

### Insights into the *C. elegans *Six family of transcription factors

The Six homeodomain protein family is one of the families of *C. elegans *transcription factors for which all the Promoterome reporter expression pattern data were determined. A deeper consideration of our data for these particular genes illustrates some of the points about reliability of the data discussed more generally above.

The Six homeodomain family members are defined by homology with *sine oculis *from *Drosophila melanogaster *(reviewed in [29]). These proteins possess a Six domain in addition to an homeodomain, both of which are essential for specific interactions both with other proteins and with DNA. Six-type proteins have been identified in species across the Metazoa, from ctenophores, cnidarians and sponges to mammals [30]. The Six family is divided into three subfamilies based on sequence similarity, Six1/2, Six3/6 and Six4/5, with genome comparisons suggesting that the last common bilaterian ancestor had a representative of all three subfamilies. Six family members are best known for their roles in eye development but appear to function in the formation of multiple head structures. *Xenopus laevis*, *Drosophila melanogaster *and mouse Six-type genes are expressed in developing anterior regions that become sensory, nervous and muscle tissue. Six family genes are also expressed in several domains outside of the head, including the gonad, stomach, and kidney and appear to play a more general role in myogenesis throughout the body axis.

*C. elegans *has four Six-type genes: *ceh-32*, *ceh-33*, *ceh-34*, and *unc-39*, all on chromosome V. *ceh-33 *and *ceh-34 *are adjacent to each other but appear to be the products of an ancient gene duplication because this genomic organization is conserved in both *Caenorhabditis briggsae *and *Caenorhabditis remanei*. Sequence comparison places *ceh-33 *and *ceh-34 *in the Six1/2 subfamily and *ceh-32 *in the Six3/6 subfamily. While *unc-39 *appears only distantly related to other Six4/5 proteins [31], the Six domain and homeodomain of human Six5 can functionally substitute for those of UNC-39 in *C. elegans *mutant rescue assays [32], strongly supporting a place for *unc-39 *in the Six4/5 subfamily.

**Figure 1:**
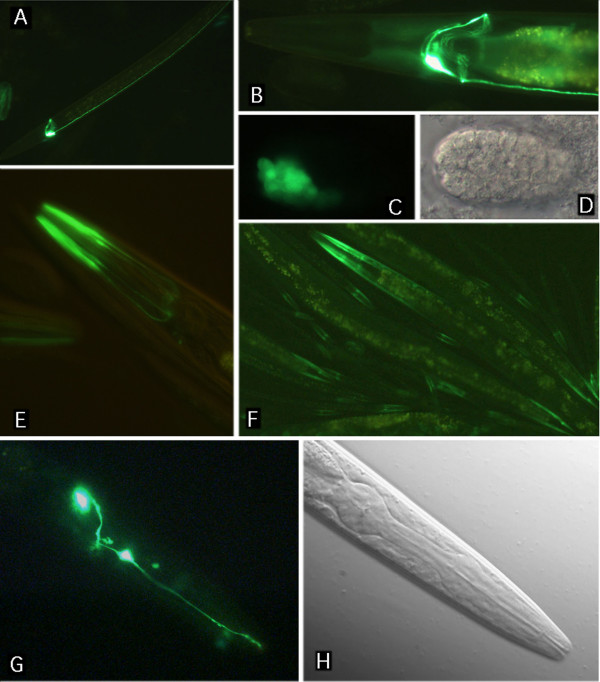
GFP expression patterns driven by Promoterome reporter gene fusions. The *ceh-32 *Promoterome insert drives GFP expression in specific nerve cells in the head of a larva (A) and adult (B), and in anterior cells of an early embryo (C), in the *C. elegans *strain UL2623. An adult's anterior body wall muscle cells express GFP under the direction of the *ceh-33 *Promoterome insert in UL1265 (E). Additional head body wall muscle cells, as well as tail body wall muscle cells, express GFP driven by the *ceh-34 *Promoterome insert in larvae and adults of UL1512 (F). The *unc-39 *Promoterome insert drives GFP expression in specific nerve cells in an adult's head of UL2387 (G). The DIC images D and H correspond to fluorescent micrographs C and G respectively. All images were captured at 200× magnification, apart from C and D, captured at 400×, and F, captured at 100×.

The Promoterome clone for *ceh-32 *contains the 1500 bp, of the 4 kb intergenic region, immediately upstream of the start codon. The GFP expression driven by this DNA fragment begins in anterior regions of the early embryo and continues through the comma stage when additional cells in the posterior start to express. The number of expressing cells then decreases until, in late embryos and all post-hatching stages, expression was seen only in one pair of head nerves (Figure 1A,B,C). The cytoplasmic localization of the GFP reveals the processes of these nerves extending ventrally to the tail, identifying these cells as one of six pairs of the AV interneurons which have very similar morphology. The expression of *ceh-32 *has previously been studied using immunostaining with a specific antibody and by creating transgenic animals expressing a functional *ceh-32::gfp *fusion [31]. Antibody staining was described as detecting CEH-32 protein in anterior regions of the gastrulating embryo and subsequent stages of embryogenesis, plus in head hypodermal cells, 24 head nerves and the somatic gonad, postembryonically. The DNA fragment fused to *gfp *in the prior work [31] was significantly different from that for *ceh-32 *in the Promoterome as it included all the exons, introns and 3.8 kb of the upstream intergenic region such that GFP was at the C-terminus of CEH-32 in the fusion protein encoded. Nematodes transformed using this larger reporter gene fusion were described as showing GFP expression in a pattern similar to that observed using the anti-CEH-32 antibody during embryogenesis and in the somatic gonad postembryonically, but with reporter expression in only a subset of the head nerves and very weak expression in head hypodermal cells. The Promoterome reporter fusion appears to have accurately revealed part of the *ceh-32 *expression pattern and gives no additional components: expression is seen in the anterior of the early embryo and remains in two nerve cells. Driving expression of unfused GFP, as when using the Promoterome inserts, reveals cell morphology, of value in nerve cell identification, that is lost in the nuclear-localized signal observed with the CEH-32::GFP fusion protein and anti-CEH32 antibody. The lack of somatic gonad expression probably reflects the absence, from the Promoterome insert, of regulatory elements needed for this expression component. There may be a similar explanation for the lack of expression in more nerve cells and in the anterior hypodermis. However, the missing elements in this case could simply be those required to maintain the expression that is present in the precursors of these cells in the early embryo. Alternatively free GFP may not be appropriately stabilized in these cells, like the CEH-32 or CEH-32::GFP might be, by interactions with other proteins and/or nuclear-localization.

The *ceh-33 *Promoterome insert consists of the entire 370 bp upstream intergenic region and drove strong GFP expression in the most anterior body wall muscles from late embryogenesis to adulthood (Figure 1E). The *ceh-34::gfp *fusion generated from the Promoterome included just 2 kb of the 3.9 kb intergenic region, from immediately upstream of the translation initiation codon, and gave strong expression in body wall muscle cells in the head and tail from late embryogenesis to adulthood (Figure 1F). GFP fusions, generated for both *ceh-33 *and *ceh-34 *using the PCR-stitching method, have been assayed previously [33]. For *ceh-33*, *gfp *was fused to the fourth exon (of six) and the fusion gene contained 7 kb of DNA upstream of the translation initiation codon, including two unrelated genes. For *ceh-34*, *gfp *was also fused to the fourth exon (again of six), but this fusion contained simply the entire 3.9 kb upstream intergenic region. Nematodes transformed with either of these fusion genes only showed weak pharyngeal reporter expression, which has been thought to be a common artifactual reporter expression domain (see above) and is in contrast to the strong, specific reporter expression driven by the *ceh-33 *and *ceh-34 *Promoterome inserts. Note that expression in the very anterior body wall muscle cells, like that driven by the *ceh-33 *Promoterome insert only much weaker, was seen unexpectedly often during the reporter expression analysis of the Promoterome and so is identified as a potential background artifact of the particular vector arrangement used here. Furthermore, the *ceh-33 *upstream intergenic region is very small, even for *C. elegans*, and although the immediately upstream and tandemly-arranged gene, C10G8.8, is not thought to be organized together with *ceh-33 *in an operon, additional regulatory elements directing *ceh-33 *expression, upstream of the intergenic region, could have been anticipated. However, in contrast to other reporter vectors, aspects of the vector arrangement used in the Promoterome analysis could have made reporter expression in these anterior body wall muscles feasible, allowing the real expression pattern of *ceh-33 *to be revealed. This interpretation is supported by the strength of the Promoterome *ceh-33::gfp *expression and the striking relatedness to the distinct expression pattern for the closely related gene *ceh-34*.

The *unc-39 *Promoterome insert is 1500 bp of the 7 kb intergenic region and drove GFP expression in the anterior of early and mid-stage embryos, and in a pair of amphids and a pair of interneurons in the head of late embryos and all postembryonic stages (Figure 1G). This expression pattern matches that observed previously, "embryonic expression" and "head neurons in larvae and adults" [34], for a larger, 2899 bp, DNA fragment from the *unc-39 *upstream region fused to *gfp *by the PCR stitching method. The expression of *unc-39 *was also previously analysed with an *unc-39::gfp *fusion [32] that had been shown to functionally complement *unc-39 *mutations. The *gfp *reporter was inserted at the end of the *unc-39 *protein-coding region within a DNA fragment containing the entire *unc-39 *locus, from 4.4 kb upstream of the translation initiation codon to the end of the transcribed region. This fusion gene was described as expressing GFP in anterior regions of the gastrulating embryo expanding with cell divisions to comma-stage embryos when some expressing cells, including Z1 and Z4 (the founders of the somatic gonad), M (the postembryonic mesoblast) and the coelomocytes [32], migrate posteriorly. In later embryos and L1 larvae GFP was restricted to ten anterior cells (mostly nerves) and the somatic gonad, with no GFP detected in later postembryonic stages. This expression pattern matched *unc-39 *mutant phenotypes which suggested roles in the development of mesodermal lineages (coelomocytes, muscles, somatic gonad) and anterior neurons (amphids, interneurons, CANs), particularly in anterior to posterior cell migration and axon guidance. Thus, both the promoter::reporter fusions and the *gfp *insertion into the gene gave similar expression patterns with one exception being that GFP of the former remained through the postembryonic stages. This difference could simply reflect decreased protein stability conferred on GFP by UNC-39, rather than a difference in transcription, as GFP expressed from a reporter gene fusion with the same 4.4 kb upstream region and the *unc-39 *3'UTR but with just the GFP ORF, also remained to adulthood [32]. The expression driven by the shorter promoter::reporter fusions also appears incomplete, these DNA fragments possibly lacking elements required for expression in the somatic gonad, body wall muscles and additional nerve cells.

Comparing GFP expression patterns driven by Promoterome inserts for all four *C. elegans *Six-type genes, to each other and with prior data, provides insight into both the functional organization of each locus and the evolution of this gene family. This example illustrates the value of the Promoterome resource and of the reporter expression pattern data.

### High expression pattern success rate and low gene duplication rate for transcription factor genes

Promoters were found to drive GFP expression for all four Six gene family members. It is notable that, in this study, a much higher proportion of reporter gene fusions yielded detectable GFP expression than in two previous large-scale experiments [35,36] utilizing reporters to examine *C. elegans *gene expression (92% compared to approximately 50%). There are many differences between the approaches used that could account for the different success rates. Genes were more likely to be targeted for inclusion in the Promoterome if they had had transcripts identified previously, the point of fusion being placed within the region known to be transcribed, and so there was already experimental evidence suggesting that most of the DNA fragments assayed here were likely to drive reporter expression. Other technical differences included the use of different reporter gene vectors and transformation of *C. elegans *by microprojectile bombardment rather than microinjection. Nevertheless, in a parallel study, using exactly the same approach as followed here, GFP expression was observed for only 42 of 58 (72%) Promoterome inserts for a set of non-transcription factor genes (J. Shingles and I. Hope, unpublished) suggesting the high success might, at least in part, relate to the focus on transcription factor genes.

*C. elegans *transcription factor genes might have qualities that mean their promoter regions are more likely to drive reporter expression. In one of the previous large-scale reporter studies [36], a major factor suggested for the low success rate for the assays was a high proportion of pseudogenes amongst the genes identified in the annotation of the *C. elegans *genome. These defective genes are a by-product of the high rates of local gene duplication, that appear to occur in the *C. elegans *genome [37], followed by genetic drift. If transcription factor genes were relatively resistant, on an evolutionary scale, to local duplication, there would be a paucity of defective transcription factor genes in the *C. elegans *genome, leading to a higher rate of success in reporter assays.

Therefore, to explore the extent of transcription factor gene duplication, the transcription factor genes of *C. elegans *and *Caenorhabditis briggsae *were compared. *C. elegans *and *C. briggsae *diverged approximately 100 million years ago [38] and the genome sequence for both has been carefully annotated, so genes duplicated since divergence of the species and retained can be readily determined. A likely *C. briggsae *orthologue was sought for each gene in the compendium of *C. elegans *transcription factor genes we generated recently [15]. The primary aim of the analysis was to identify *C. elegans *genes that were likely to have arisen by duplication on the lineage to *C. elegans *and therefore a table of the closest *C. briggsae *orthologue, for each *C. elegans *transcription factor gene, matched to its closest *C. elegans *gene was generated [See additional file 3]. Where a *C. elegans *transcription factor gene lacks a *C. briggsae *orthologue that gene is likely to have arisen through local duplication. The alternative explanation, that the *C. briggsae *orthologue has been lost, was typically rendered even less likely when the molecular phylogenetic relationships within a family were evaluated.

**Figure 2:**
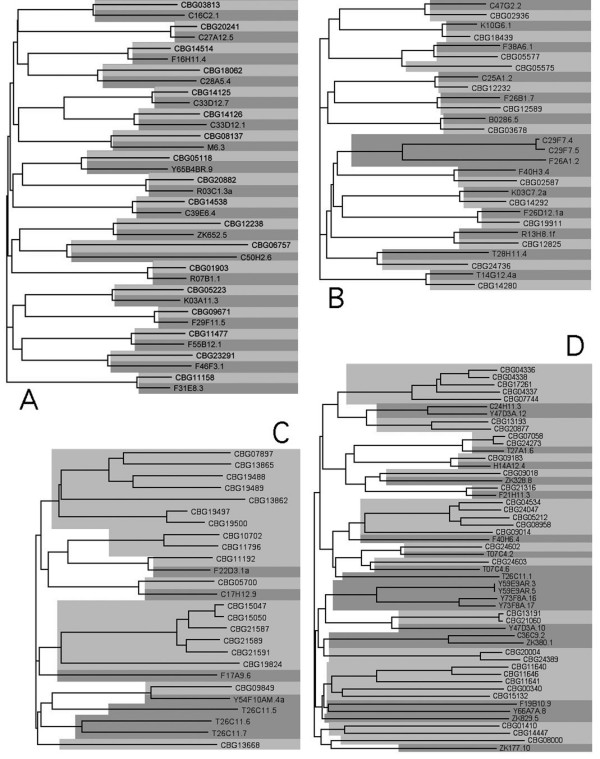
Representations of the molecular phylogenies of the *C. elegans *and *C. briggsae *members of the NK class of homeodomain (A), the forkhead (B), the CUT class of homeodomain (C) and the T-box (D) transcription factor gene families. *C. briggsae *genes are shaded in light grey and *C. elegans *genes are shaded in mid-grey.

For many transcription factor gene families there was an exact, or close to exact, one to one match between *C. elegans *and *C. briggsae *orthologues (Table 2). This is in sharp contrast to the approximately 65% of genes genome-wide that can be placed in orthologous pairs in comparisons between these two species [38]. A molecular phylogeny for the NK class of homeodomain containing transcription factors (Figure 2A), one of the families with a perfect correspondence between *C. elegans *and *C. briggsae *orthologues, reveals the strength of confidence in the orthologies.

For some transcription factor gene families a few exceptions to the perfect orthologue correspondence were found which probably do not actually correspond to duplication of transcription factor gene function. The forkhead transcription factor gene family molecular phylogeny (Figure 2B) reveals one *C. briggsae *gene and three *C. elegans *genes lacking an orthologue in the sister species. The *C. briggsae *gene CBG05575 has resulted from a tandem duplication of CBG05577, the orthologue of the *C. elegans *forkhead gene F38A6.1 (*pha-4*), but is probably a defective copy. There is a stop codon and a frameshift in the region of CBG05575 encoding the forkhead DNA binding domain that would prevent production of a functional transcription factor. The increased sequence divergence for this pseudogene, as a result of relaxation of selection, along with the problem of gene structure prediction, has meant that the branch leading to CBG05575 in the molecular phylogeny appears misleadingly much deeper than it probably should be. The three *C. elegans *genes that lack *C. briggsae *orthologues, C29F7.4 (*fkh-3*), C29F7.5 (*fkh-4*) and F26A1.2 (*fkh-5*), are all related to each other, the former two being a tandem gene pair resulting from a very recent single gene duplication event. The length of the branches in the molecular phylogeny leading to these three genes are also longer than for any other members of this gene family suggesting relaxation of selection as a result of loss (or change) of function. Furthermore, promoters for these three genes drove reporter expression in all somatic cells in the early embryo [39], an odd expression pattern for a transcription factor that is expected to function in distinguishing between developmental cell fates. These observations could suggest that these three *C. elegans *forkhead genes may no longer encode functional regulatory transcription factors.

A few transcription factor gene families, however, showed a marked low proportion of clear orthology between *C. elegans *and *C. briggsae *members (Table 2). The CUT class of homeodomain containing transcription factors (Figure 2C) and the T-box family (Figure 2D) are clear examples of where genes have undergone repeated duplications since the divergence of *C. elegans *and *C. briggsae*. This reduces confidence in the orthologies that have been assigned and the level of full functional correspondence between genes of these families in the two species may be even lower than presented. Three of the four *C. elegans *CUT homeodomain encoding genes that lack a *C. briggsae *orthologue result from the tandem amplification of one gene and are more closely related to each other [40] than the molecular phylogenetic tree suggests. However, this gene family has undergone extensive amplification in the *C. briggsae *lineage. The T-box gene family has undergone even more extensive duplications and in both the *C. elegans *and the *C. briggsae *lineage. Therefore, while on average transcription factor genes show a lower frequency of gene duplication for these *Caenorhabditis *species, as noted for other organisms [41], this is not true for all transcription factor gene families.

## Conclusion

The Promoterome resource was generated as an important experimental subject through which an understanding of the control of expression of the *C. elegans *genome can be constructed [8]. The Promoterome is a characterized bank of clones of the regions immediately upstream of the start of *C. elegans *genes, regions containing the highest density of the *cis*-acting elements that control gene expression. Results from assays of different aspects of promoter activity can be directly related to each other when the assays have been applied to identical DNA fragments, as cloned in the Promoterome. Approaches, such as the yeast one-hybrid assay, will reveal transcription factors which can bind to each promoter [14,42]. To relate this to promoter function *in vivo *the pattern of expression driven by these DNA fragments needs to be determined and this can be revealed directly in *C. elegans *strains generated by transformation with Promoterome inserts fused to reporter genes, as described here. These data will be essential for construction of computer simulations representing how transcription of the entire genome is coordinated. We have focused initially on promoters of transcription factor genes as these will be at the heart of such models. A final set of data, essential for construction of these models and complementary to the other data sets, is the distribution of the transcription factors themselves and approaches to deliver these data, that can be scaled up, are now being developed [9]. Further technical advances, to allow automatic determination of *in vivo *distributions of transcription factors and promoter activity, to single cell resolution, on a large scale [43], will also be crucial.

Transcriptional control elements are not found exclusively in the region immediately upstream of the start of a eukaryotic gene and therefore Promoterome inserts will not contain all transcription factor binding sites of relevance to every gene's expression. Furthermore, in the transgenic *C. elegans *strains generated the reporter gene fusions are not in their natural context. Therefore, we have evaluated how accurately the expression patterns driven by Promoterome inserts report on the transcriptional activity of a gene's promoter by comparing our expression patterns with those reported by others examining gene product distributions by immunofluorescence microscopy or *in situ *hybridisation.

With the experience of examining hundreds of reporter gene expression patterns in *C. elegans*, the Promoterome inserts provide a reliable report on a gene's transcriptional activity. Subtle differences in timing between previously described gene product distributions and the promoter::reporter fusion gene expression pattern can be ascribed simply to posttranscriptional events and are not an issue. Certain expression pattern components, seen with more reporter fusion genes than expected based on other expression pattern data and which may result from cryptic enhancers in the cloning vector only revealed according to properties of the particular promoter being assayed, do need to be treated with scepticism but are readily recognised in large scale studies. For some promoter::reporter fusion genes components of complex expression patterns appear to be missing, but this may be due, although clearly not in all cases, to a gene having alternative promoters, the expression observed accurately reflecting the transcriptional activity of the promoter assayed. Once the repeatedly-seen, background expression pattern components are dismissed, 90% of the expression pattern data generated with Promoterome reporter fusion genes is reporting accurately on transcriptional activity of *C. elegans *gene promoters.

The *C. elegans *Six family is one of the families of transcription factor genes for which we have generated expression pattern data for all members, using the Promoterome resource. These genes' expression patterns appear related to those observed for Six-type genes in other animals: sensory, nervous and muscle structures of the head, as well as gonad tissues and other muscles outside the head. This suggests that the roles played by genes of the Six family in flies, frogs and mammals are shared by nematodes. The strong and specific expression for the promoters of the adjacent Six family genes *ceh-33 *and *ceh-34*, reveals overlapping expression in head muscles for a gene pair that has been retained in all species of the *Caenorhabditis *genus for which the genome has been sequenced. There is no obvious similarity in nucleotide sequence between the Promoterome inserts of these two genes, but degeneracy and small size of transcription factor binding sites means that the overlap in their expression patterns could still be driven by the same transcriptional control system. Retention of these two genes, and presumably the overlap in their expression patterns, over considerable evolutionary time suggests important functions for both in the very anterior head muscles. Although specific knockdown of *ceh-33 *and *ceh-34 *individually, by RNAi, generated no obvious phenotype [4], closer attention to movement of the tip of the head in single and double knockdown experiments may reveal synergistic or overlapping function for these two genes. The more extensive expression of *ceh-34 *in the head as well as in the tail suggests a unique function for this gene in additional muscle cells that may also be revealed by closer inspection of *C. elegans *subject to *ceh-34 *RNAi. All body wall muscle cells appear very similar morphologically and there is no information yet on how or why the very anterior and posterior body wall muscle cells are distinguished from more central body wall muscle cells.

The tandem gene pair of *ceh-33 *and *ceh-34 *appears to be one example of the relatively rare event of a transcription factor gene that has been locally duplicated with both copies retaining function. The study of transcription factor gene duplication through identification of orthologues between *C. elegans *and *C. briggsae *was initiated because of the high rate of success of reporter gene expression for Promoterome inserts assayed here. In a previous study using the reporter approach [36] the poor success rate specifically for recently duplicated genes had led to the suggestion that there were many pseudogenes amongst the annotated *C. elegans *genes. This large number of non-processed pseudogenes would be a consequence of a high local gene duplication rate within the *C. elegans *genome, with subsequent drift of extra gene copies. In contrast, observation of GFP expression of reporter fusions for almost all transcription factor gene promoters would suggest there are few pseudogenes, as a consequence of low local duplication events, specifically for this type of gene.

Subsequent evidence further supports there being an abundance of dysfunctional genes in the *C. elegans *genome. High rates of gene duplication, with genetic drift of duplicated genes, may be a consequence of reproduction principally by self-fertilization. Over a thousand pseudogenes are now specifically annotated as such in WormBase [44]. In a large scale RNAi study [4], recently duplicated genes yielded detectable phenotypes less frequently than other genes and, although interpreted in terms of overlapping function for recently duplicated genes, this would also be consistent with many recently duplicated genes no longer retaining any function. Nonsense-mediated decay of non-coding transcripts could mean that there is less pressure on dysfunctional genes to be deleted from the genome and so pseudogenes may remain longer, thereby accumulating in a genome in larger numbers than might have been expected [45]. Genes involved in interacting with *C. elegans' *natural environment appear to undergo frequent duplication events, driven by their ecological interactions, and such gene families are consequently large, showing relatively high rates of gene birth and death [46]. There are many members of these gene families inactivated by mutation in the researcher-defined wild-type strain, N2, while functional in other, independently-isolated wild-type strains and, therefore, the species [46]. Such genes, although included within the large proportion of the genome identified as dysfunctional and therefore pseudogenic in an earlier study [36], should not be designated as pseudogenes [46].

Transcription factor genes, in general, appear excluded from this high rate of local gene duplication within the *C. elegans *genome. Similar observations were made for *Arabidopsis thaliana *[41]. In this plant, in addition to a high rate of local gene duplication like *C. elegans*, the genome has undergone three recognizable whole genome duplication events revealing that transcription factor genes have duplicated specifically through the latter rather than the former mechanism. Furthermore, the *Saccharomyces cerevisiae *genes that decreased fitness when over-expressed were enriched for transcription factor genes [47]. For many of the *C. elegans *transcription factor gene families, the high confidence in and completeness of *C. elegans*/*C. briggsae *orthologies (and beyond (data not shown)) suggests these gene families have been fixed in extent for far longer than the time since the *C. elegans*/*C. briggsae *divergence. Given the multiplicity of many transcription factor genes' roles, with distinct gene expression domains, it might have been anticipated that such genes would be the ideal subjects for local gene duplication, with rapid sub-functionalization of the duplicated copies. However, transcription factor genes, that have been duplicated, can retain overlapping function over an evolutionary time-scale [48]. Perhaps the complexity of transcription factor gene expression patterns, matched by the complexity of their transcriptional control with elements for different expression domains tightly intermingled, means that simultaneous separation of different functions during gene duplication is impossible. Although experimental evidence, for example from chromosomal rearrangements, suggests extra copies of transcription factor genes in general are not deleterious in *C. elegans *our observations suggest that on an evolutionary scale the function of these genes is exquisitely sensitive to dosage.

However, it would be wrong to suggest that all transcription factor genes are resistant to local gene duplication. In fact, the high success rate for reporter gene expression that initially led to this consideration in part reflects which transcription factor gene families we focused on. A few transcription factor gene families show relatively high rates of gene duplication since the *C. elegans*/*C. briggsae *divergence. The nuclear hormone receptor transcription factor gene family is very large with just 55% of *C. elegans *genes having *C. briggsae *orthologues. These qualities, along with the steroid binding domain for sensing levels of hydrophobic compounds, would be consistent with many members of this family being involved in environmental sensing and gene duplication in this gene family being driven by ecological interactions. Most transcription factor gene families are involved in directing development. As *C. elegans *development is largely invariant and does not change substantially with the environment, ecological factors would not drive duplication of members of such transcription factor gene families. The CUT homeodomain and the T-box transcription factor families have the lowest level of *C. elegans*/*C. briggsae *orthology. Curiously, the *Drosophila melanogaster cut *gene, the founding member of the CUT family, is involved in external sensory organ development [49] and so perhaps duplication within this transcription factor family is also driven by ecological interactions. Several of the Promoterome inserts for *C. elegans *CUT family members drove reporter expression in sensory nerve cells. Members of the *C. elegans *CUT and T-box transcription factor gene families do not appear as sensitive to increased gene dosage resulting from local duplication as members of other transcription factor gene families.

Work with the Promoterome resource has focussed initially on transcription factor genes anticipating that these would form the base of any systems biology model of genomic regulation. Transcription factor genes with *C. briggsae *orthologues are more likely to be involved in developmental control processes and hence to be at the core of these models.

## Methods

### Generation of promoter::reporter fusions

Promoterome clones and promoter::*gfp *fusions were generated as previously described [8]. Briefly, Gateway-compatible primers designed to target regions upstream of *C. elegans *ORFs were used in PCRs with *C. elegans *(wild-type N2 strain) genomic DNA, with the resulting product cloned into the MultiSite Gateway Entry vector pDONR P4-P1R using BP clonase. The promoters were moved from the Promoterome Entry clone to the MultiSite Destination vector pDEST-DD04 using LR clonase, such that the translational start codon of the *C. elegans *gene would lead into the appropriate translational reading frame of the reporter. The reporter contains artificial introns and the *C. elegans unc-54 *3'UTR to improve expression levels in *C. elegans*. The vector also contains a wild-type *C. elegans unc-119 *gene to permit selection of transgenic animals. Both the initial cloning and fusion generation steps were performed in 96-well plates. Initial confirmation of each clone was by PCR with vector-specific primers. To ensure each Promoterome insert was correct, before *C. elegans *transformation, every fusion underwent either diagnostic restriction enzyme digests and/or DNA sequencing.

### Transformation of *C. elegans *by microprojectile bombardment

Procedures for transformation by bombardment were developed from a protocol, provided by R. Andrews and J. Ahringer (personal communication), derived from [16]. Large scale cultures of *C. elegans *strain DP38 (*unc-119 (ed3)) *were used for transformation by bombardment. Nematodes from five, 5 cm NGM agar plates, were washed into a flask containing 100 ml S-basal (0.1 M NaCl, 0.05 M Potassium Phosphate pH6, 5 μg/ml cholesterol (from a 5 mg/ml stock in ethanol)) supplemented with 50 μg/ml Nystatin (Sigma-Aldrich, UK.), 50 μg/ml Streptomycin (Sigma-Aldrich, UK.). An *E. coli *HB101 bacterial suspension was prepared, by centrifugation of a 1 litre overnight culture grown in LB medium and resuspension of the cells in 6 ml of S-basal, and 4 ml of this suspension was added to each flask, one flask per plasmid. The nematode culture was incubated, with shaking, at 20°C, for 7 days and then transferred into large tubes for the nematodes to settle out, at room temperature, under gravity over 10 minutes. The supernatant was transferred to a separate tube and allowed to settle a second time, but for 15 minutes and on ice. Nematodes in the first pellet, mainly adults and L4s, were used for bombardment immediately. Nematodes in the second pellet, mainly young larvae, were used to inoculate a flask with a further 100 ml of S-basal plus antibiotics and bacteria. These secondary flasks were incubated for three or four days, until the culture had almost cleared, when nematodes were harvested as before. This protocol allows continuous culture, providing nematodes suitable for bombardment every 3 to 4 days. New cultures were added and older cultures were removed on a weekly basis.

Approximately 7 μg of each plasmid (Qiagen plasmid DNA mini-preparation) was linearised by digestion with *Ngo*MIV, *Hin*dIII or *Bam*HI restriction enzyme in standard buffers (NEB) in a total reaction volume of 35 μl before precipitation onto gold particles for bombardment. 60 mg gold particles (0.3–3 μm, Chempur, Germany) were added to 2 ml 70% ethanol, vortexed for 5 minutes, allowed to soak for 15 minutes and then washed three times with sterile water. The final gold pellet was resuspended in 1 ml of sterile 50% glycerol. 30 μl of the plasmid digest was added directly to 70 μl of gold bead suspension and vortexed for 1 minute. 300 μl of 2.5 M CaCl_2 _was then added drop wise and while vortexing to prevent sedimentation of the particles. 112 μl of 0.1 M spermidine was then added in the same manner. Following vortexing for 5 minutes, the suspension was centrifuged at 6000 rpm for 5 seconds and the supernatant discarded. The gold beads were then washed in 800 μl 70% ethanol and finally resuspended in 70 μl 100% ethanol with continued vortexing until ready to use.

For each bombardment using the Bio-RadPDS-1000/He with Hepta adapter, a 9 cm NGM agar plate seeded with OP50, was inoculated with seven 150 μl aliquots of the sedimented nematodes, placed in positions corresponding to the target of the Hepta adapter. The bombardment procedure was then followed according to the manufacturers instructions. The inoculated NGM agar plate was placed on the second target shelf up and 9.3 MPa (1350 psi) rupture disks were used with a vacuum of 91 kPa (26 in. Hg). Following bombardment, 1 ml of M9 buffer was added to each plate and the nematodes were left to recover for 1 hour at room temperature. Nematodes were then washed from the plates with 4 ml M9 buffer and 0.5 ml of the nematode suspension was used to inoculate each of seven seeded 9 cm NGM plates. All eight plates, including the plate used in the bombardment, were incubated at 20°C for three weeks.

Plates were then examined for the presence of animals rescued for the *unc-119 *mutant phenotype, i.e. those having survived starvation through their ability to form dauers and showing wild-type movement. Four individuals from each large plate were transferred individually to seeded 5 cm NGM agar plates. After 7 days the established lines were assessed for level of transmission of the rescued phenotype and the transgene. The plate with the highest level of transmission was retained and the remaining three were discarded. Up to eight independent lines were generated per bombardment in this manner. Nematode lines likely to contain integrated transgenes were identified from the absence of *unc-119 *mutant progeny over several generations.

Transformation by microinjection was as previously described [10] with pRF4 and the plasmid containing the GFP fusion injected as a mixture, both at 100 μg/ml, and transformed lines established and maintained by screening for individuals with the roller phenotype.

### Reporter expression pattern characterization

The target of eight independent transgenic nematode lines, for each reporter gene fusion, was typically met and all lines generated were examined for GFP expression. Populations of hermaphrodites, at all stages of development, were examined by fluorescence microscopy with Chroma Technology Corp. filter set 41012 on a Zeiss Axioplan microscope equipped with DIC optics. Spatial and temporal GFP expression patterns were determined for each transgenic nematode line, to the cellular level where straightforward. Photomicrographs, representative of the expression pattern observed, were collected with a Photometrics CoolSNAP camera and Improvision Openlab software. Data were incorporated into the Hope Laboratory Expression Pattern Database [18]with details of the DNA fragment assayed, a text description of the GFP expression pattern, example images and further information about each strain generated. Typically, three lines, those that showed the highest level of transmission and/or GFP expression, were retained, including any lines with the introduced DNA chromosomally integrated. Frozen stocks were prepared for each retained strain to provide a permanent accessible resource for future reference.

### Identification of transcription factor gene orthologues

*C. briggsae *genes [38] orthologous to *C. elegans *transcription factor genes [15] were extracted from WormBase (freeze WS150) [44]and from downloaded Inparanoid [50] data [51]. Blast analyses [52] were used to identify additional family members in the *C. briggsae *genome sequence data and to confirm or refine evolutionary relationships. Potential molecular phylogenies were generated using ClustalX version 1.8 [53] to align sequences through the Neighbour-joining method with output of unrooted trees for examination in Treeview PPC version 1.6 [54]. The analysis was only taken far enough to be confident of relationships between *C. elegans *and *C. briggsae *orthologues, and relationships implied by deeper branches will be of lower significance.

## Authors' contributions

JSRH and JS generated and interpreted the majority of the reporter expression patterns and drafted the manuscript. DD generated the Promoterome resource and the reporter gene fusions. CAG and IAH generated and interpreted some of the expression patterns. The deeper consideration of the SIXHD data was carried out by JSRH. IAH carried out the *C. elegans/C. briggsae *gene comparison. MV, AJMW and IAH conceived, instigated and co-ordinated the project. All authors contributed to, read and approved the final manuscript.

## Supplementary Material

Additional File 1 contains descriptions of GFP expression patterns, in terms of tissue type and life stage, as driven by Promoterome inserts for the 366 transcription factor genes assayed. Click here for file

Additional File 2 contains a summary of comparisons between Promoterome driven reporter gene expression patterns and expression patterns generated previously by immunomicroscopy or *in situ *hybridization for the 40 *C. elegans *genes for which such comparison can be made. Click here for file

Additional File 3 contains a list of the 934 putative transcription factor genes, wTF2.0, with their *C. briggsae *orthologue where an orthologue could be identified. Click here for file

## Figures and Tables

**Table 1:** Summary of reporter expression pattern data gathered for Transcription Factor gene Promoterome inserts.

		Number of Genes	Percentage of Genes Assayed
	Total Genes Assayed	366	100%
	No Expression	31	8%
	Ubiquitous Expression	31	8%
Expression in the:	Nervous System	246	67%
	Muscle Cells	129	35%
	Digestive Tract	178	49%
	Excretory Cell	27	7%
	Gonad	42	11%
	Hypodermis	31	8%
	Vulva	37	10%
	Seam cells	31	8%
	Coelomocytes	33	9%
Life stages showing expression	Adults	323	88%
	Larvae	327	89%
	Late Embryogenesis	294	80%
	Mid Embryogenesis	159	43%
	Early Embryogenesis	97	27%

**Table 2:** The proportion of *C. elegans *genes, with a *C. briggsae *orthologue, in each transcription factor gene family.

Transcription factor family	Number of *C. elegans *genes	Number of *C. elegans *genes with *C. briggsae *orthologues	Percentage of *C. elegans *genes with *C. briggsae *orthologues
HD – NK	18	18	100
HD – HOX	14	14	100
HMG box	13	13	100
HD – LIM	7	7	100
HD – SIX	4	4	100
ZF – FLYWCH	4	4	100
WH – TDP	3	3	100
ZF – THAP	3	3	100
HD – PRD	12	11	92
ZF – C2H2 – 3 fingers	34	31	91
ZF – C2H2 – 5 fingers	11	10	91
CBF	9	8	89
ZF – DHHC	15	13	87
MH1	7	6	86
ZF – CCCH	7	6	86
bHLH	37	31	84
ZF – BED	6	5	83
ZF – C2H2 – 4 fingers	16	13	81
bZip	31	25	81
WH – Fork Head	15	12	80
WH – ETS	10	8	80
COLD BOX	5	4	80
HD – TALE	5	4	80
ZF – C2H2 – 7 fingers	5	4	80
ZF – DM	9	7	78
MADF	8	6	75
AP-2	4	3	75
HD – POU	4	3	75
ZF – C2H2 – 1 finger	80	59	74
ZF – C2H2 – 2 fingers	33	24	73
MYB	7	5	71
ZF – GATA	10	7	70
AT Hook	13	9	69
ZF – C2H2 – 6 fingers	13	9	69
ZF – NHR	272	150	55
WH	4	2	50
T-box	21	10	48
HD – CUT	7	3	43
HD	12	4	33
